# Technology scripts in care practice: A case study of assistant nurses’ use of a social alarm system in Swedish nursing homes

**DOI:** 10.1177/20552076221089077

**Published:** 2022-03-24

**Authors:** Fangyuan Chang, Sanna Kuoppamäki, Britt Östlund

**Affiliations:** Department of Biomedical Engineering and Health Systems, School of Engineering Sciences in Chemistry, Biotechnology and Health, 7655 KTH Royal Institute of Technology, Stockholm, Sweden

**Keywords:** eHealth, general, delivery of healthcare, information systems, acceptance, qualitative, studies

## Abstract

**Background:**

Technologies such as social alarm systems contain expectations about how they should be integrated and used in practice. These expectations, also called technology scripts, usually fail to consider all the complexity in care practice. Shifting the focus from technology scripts to care practice, this paper examines how a social alarm system is used in assistant nurses’ care practices in nursing homes.

**Methods:**

The paper draws on observations of assistant nurses’ daily tasks (32 h) and semi-structured interviews with assistant nurses (*n* = 12) in two Swedish nursing homes. The observation data were used to understand the care contexts and assistant nurses’ technology-mediated care practices, while interviews were used to deeply understand assistant nurses’ perceptions of the system, their care practices, and which aspects they considered during the provision of care.

**Findings:**

We show the complexities involved in integrating a social alarm system into care practices based on assistant nurses’ situational and personal interpretations of both technology scripts and quality of care. The technology-mediated care practices consist of receiving alarms from residents, checking alarms via alarm phones, responding to alarms via alarm phones, checking specific residents’ situations in person, documenting all finished alarms, and documenting some finished alarms. In these practices, the assistant nurses defined technology scripts according to their expected requirements and outcomes, and meanwhile considered the quality of care by evaluating the priority of practical, moral or relational care in the situations at hand. Through further negotiations with the defined scripts and the considered quality of care, the assistant nurses decided on the final way of following (or not following) specific scripts in practice.

**Conclusion:**

Results from our study portray the complexity of technology in care practices. The findings contribute to increased understanding of technology-mediated care practices in nursing homes, and research on technology scripts in institutional settings.

## Introduction

Many healthcare organizations, ranging from nursing homes to hospitals, are implementing new technological solutions to improve the quality of care.^1^ Along with this, however, extensive studies have highlighted the mismatch between the promises of technology's effects and the actual outcomes.^2–4^

The primary reason for this mismatch is that technologies contain expectations of how they should be integrated and used, while these expectations usually fail to consider all the complexity in care practice. Specifically, when designing a technology, designers and engineers tend to anticipate how a technology should function according to their interpretations of user needs and user interactions with the technology. In this way, technologies contain prescriptions resembling scripts that show how users should relate to, adapt and interpret the technology.^5,6^ However, given the complexity of reality, these technology scripts often fail to include all the potential aspects relating to technology use. The complexity is compound by different interpretations, such as what to think of the technology, how to use the technology and how to perceive the use of the technology. In other words, the use of technology is influenced by individual arrangements, and negotiates with these arrangements in care practice.^7–9^ In this view, the pre-existing practices and the technology scripts affect and intertwine with each other, co-contributing to a new practice in organizations.^9–13^ Hence, technology scripts affect but do not determine the actual use of technology. What is determinative is not technology scripts and the expected outcomes, but rather the lived reality of technology use in care practice.

Shifting the focus from technology scripts to care practice, there is a long tradition of research which reveals factors affecting the integration of technology into practice, and unanticipated consequences compared to expected outcomes.^14–16^ However, the question of how technology-mediated care practices are being delivered as practical accomplishments for care provision has been largely unexamined.^17,18^ A research gap still remains concerning the process through which technology is used in care practices, and healthcare professionals’ considerations of the objectives of care provision during the process. In other words, we lack empirical and theoretical understanding of how healthcare professionals incorporate technology scripts in care practices, with consideration to the quality of care provision.

In this study, we examined the characteristics of technology scripts in care practices, healthcare professionals’ approaches to incorporating the scripts of technology into their care practices, and the relationship between their approaches and the quality of care. To understand these aspects, we conducted a fieldwork within a five-month period on the use of a social alarm system by assistant nurses in two nursing homes in Sweden. In the following sections, we first provide a general introduction to social alarm systems and their scripts and follow this with a discussion of care provision in nursing homes and the complexity of care practices. We then describe the methods used in the study and conclude by discussing and elaborating on findings.

## The social alarm system and its scripts

Social alarm systems, a widely accepted technology in nursing homes in Sweden, allow residents to summon help from their assistant nurses remotely.^19^ Although the system is designed for urgent situations such as if a resident has fallen, its use contexts also include situations where residents ask for help in terms of daily activities such as toileting, dressing and eating. The system is usually comprised of alarm phones for assistant nurses, pendants/wristbands with alarm buttons for residents and a signal delivery transmitter. Although the devices are not all designed in exactly the same way, the systems are commonly aimed at transmitting alarms to a designated contact.

**Figure 1.**
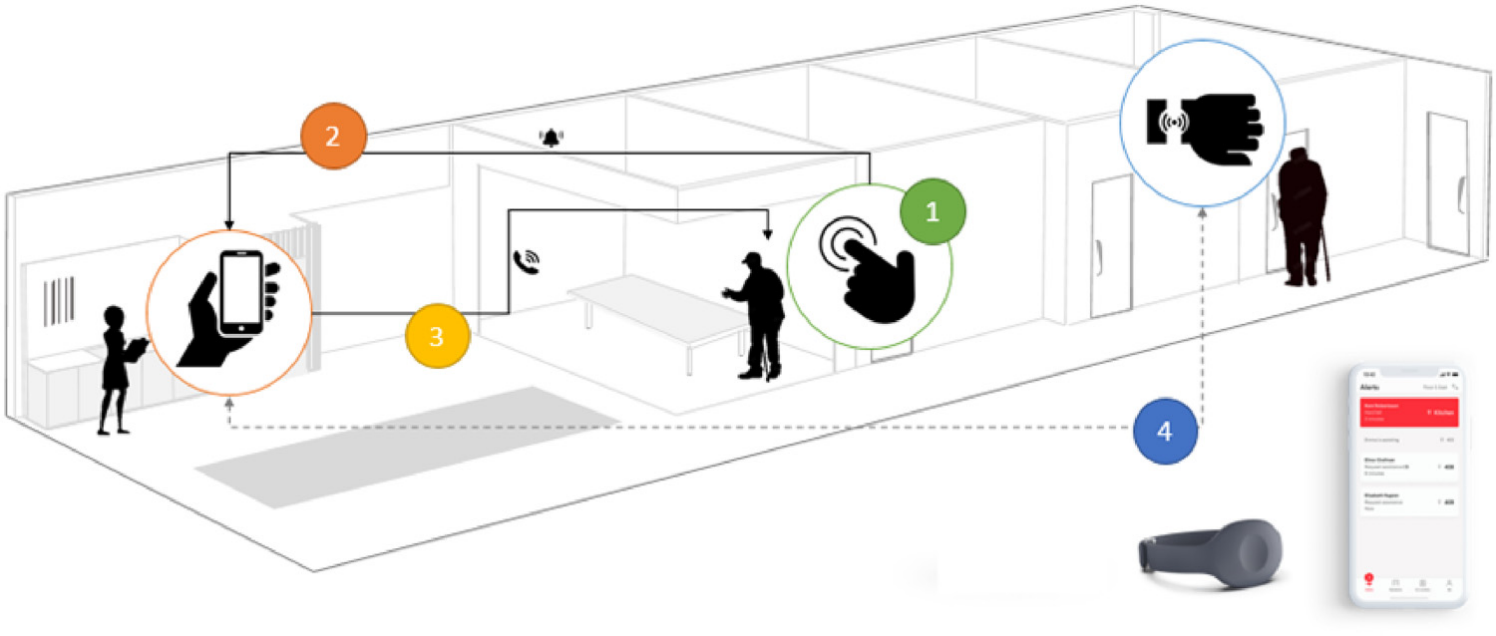
Social alarm systems in nursing homes.

The system in our study consists of wristbands with buttons worn by the residents of the two nursing homes, and an app that is installed in the alarm phones of all assistant nurses (Figure 1). When a resident needs assistance, he or she can send out alarms by clicking the alarm buttons, and assistant nurses can connect with the resident by answering the received alarms on their phones.

The scripts of the social alarm system include alarm getting activated, notifying designated contacts, enabling remote communication and tagging finished alarms (Figure 1). Specifically, when residents click their alarm buttons, the system generates and delivers alarm signals to designated alarm phones. As the signals successfully arrive at the alarm phones, assistant nurses receive alarm notifications with information such as who sent the alarm and at what time. They can then use the alarm phones to communicate with specific residents about their needs and take further actions. The alarms are tagged as ‘finished’ by assistant nurses when they finish handling the care cases corresponding to the alarms. The information in these alarms (e.g. who sent the alarms, which alarms have been finished) is synchronized in all alarm phones automatically. In this view, the system enables strong social network connectedness between residents and assistant nurses. Its service is thus seen as a representation of person-centredness.^20,21^

In exploring the incorporation of technology scripts, however, current studies primarily take the perspective of older adults to describe how technology is used.^22,23^ Technology use by healthcare professionals such as assistant nurses in nursing homes has received significantly less attention. In a few studies which have noticed this gap, research reveals that healthcare professionals play an important role in technology use, as they connect technology with older adults in the care services.^11,18,24^ These studies suggest that a deep understanding of healthcare professionals’ technology-mediated practices is still lacking and thereby call for more empirical research.

## Care provision in nursing homes

Care provision is an unavoidable theme in any attempt to understand healthcare professionals’ practices. Many researchers define care as a relational, moral and practical concept.^25–27^ In this vein, care provision is described as ‘experience-based actions developed through the three central contradictions: care as an orientation towards others, care as a moral strain and care as a technique’.^26^ Specifically, relational care means providing care and concern to others. Hence, patient-leading care and person-centred care belong to this aspect. Moral care refers to the notion that healthcare professionals treat everyone fairly and respectfully and understand situations in their working environment. It thus relates to moral care when care provision is just or ethically legitimated. Lastly, practical care is related to the concrete measurement of care provision. In this aspect, care is provided as practical care when it is cost-effective. According to the findings of various studies, healthcare professionals characterize patient care as meaningful and satisfactory when they are able to balance these three seemingly contradictory aspects of care.^28,29^

Although the categories of care are clear, their application in care practices is hardly determined in advance.^11,13^ As various scholars suggest,^30–33^ ‘care is not possible when the practitioners care more about adhering to the rules and procedures than they do about doing the right thing’. This is consistent with what Mol has emphasized: care provision relies on trying out different approaches and identifying what works in each situation.^30,34^ Therefore, care provision should be understood as a response to situational circumstances.

## Technology-mediated care practices

Reflecting on the technology scripts and the care provision in practice, we understand that healthcare professionals’ technology-mediated care practices take on meanings in relation to the technology, individual actions and approaches of care provision. In other words, technology-mediated care practices can be viewed as the inter-relational achievements of healthcare professionals themselves, as well as the technology scripts and care concepts they use. Consequently, studies concerning technology use in care should not start from a pre-conceived idea of how technology should be used and how care should be provided, but should instead be sensitive to how technology is actually used for providing care in specific situations.

The question then arises of how healthcare professionals from the technology-mediated care practices in specific situations. Only a few studies have investigated this question. Mort et al.^11^ illustrated how the introduction of technology brings new activities, within which healthcare professionals subvert the expected uses in particular contexts. Oudshoorn^24^ shows how healthcare professionals manage new situations in which technology delegates work that was traditionally performed by themselves to patients. Saborowski and Kollak^18^ demonstrated how healthcare professionals perceive technology to be in competition with important aspects of care as they meet technical, legal and financial constraints during technology use.

These studies have investigated healthcare professionals’ technology-mediated practices in a variety of care cases, and have found that healthcare professionals meet great challenges in such services due to the complexity of technology in care practices. Yet they stopped short of really trying to understand the specificities of the connection between healthcare professionals’ use of technology, the technology scripts, and the care provision in such care services. To fill this gap, thorough on-site studies are necessary, with a particular focus on the technology-mediated care practices in relation to the technology scripts and different care concepts. This study aims to understand this through a case study of assistant nurses’ use of a social alarm system in nursing homes.

## Methods

### Data and participants

The study reports findings from observations and semi-structured interviews conducted in two nursing homes in Sweden. One nursing home was located in an urban area, and the other in a rural area. The social alarm system in the two nursing homes is the same. Both nursing homes included special care units for patients with dementia and general units for older adults with other disabilities. Each nursing home had approximately 55 residents.

Empirical data, collected between August and December 2019, includes observations of assistant nurses’ daily tasks (32 h) and semi-structured interviews with assistant nurses (*n* = 12). All participants in this study are assistant nurses, a title which in Sweden refers to qualified professionals with a three-year training degree. They are responsible for the primary provision of care in Swedish nursing homes and are assisted in their work by nurses’ aides, who often have extensive care experience but less formal training.^35^

### Recruitment of participants

Assistant nurses were recruited based on their informed consent and interest in participating in the study. A questionnaire was sent to all assistant nurses in each nursing home. For inclusion in the study, participants needed to be assistant nurses, proficient in English, and use the social alarm system to provide care in their daily work. To include assistant nurses from different socio-demographic backgrounds, the following variables were also considered in the recruitment process: gender, employment type, elderly care experience, and social alarm system experience. A total of 12 assistant nurses were ultimately recruited (Table 1).

**Table 1.** Participant variables (*N* = 12).

Variables	Number
Region	
Urban	8
Rural	4
Gender	
Female	9
Male	3
Employment type	
Part-time	2
Full-time	10
Years in elderly care	
0–5	3
5–10	7
10 +	2
Years of social alarm system use	
0–5	4
5–10	6
10 +	2

### Data collection

Data were collected through observation and interviews to enable a more complete picture of assistant nurses’ technology-mediated care practices.^36^ The observation data were used to understand the care contexts and assistant nurses’ technology-mediated care practices, while interviews were used to deeply understand assistant nurses’ perceptions of the system, their care practices, and which aspects they considered during the provision of care.

All observations took place in the public spaces in each nursing home, twice a week over the course of two months. The observations were structured to ensure well-organized field notes concerning how the assistant nurses provided care with the support of the social alarm system.

All interviews were audio-recorded and transcribed. Interviews followed a discussion template concerning assistant nurses’ perceptions of the system, their care practices, and considered aspects during the care provision. The author asked interview questions flexibly in order to allow assistant nurses to present their knowledge according to their own specific occupational context and experience. The records ranged in length between 12 and 60 min depending on the assistant nurses’ working experience and knowledge. Assistant nurses who had limited knowledge of using social alarm systems or limited experience in elderly care had shorter interviews. During the interview, regular summaries were used to ensure the validity of the collected data. Saturation was used as a stopping criterion: data collection ended when no major new insights had emerged in three consecutive interviews. All interviews were conducted in a private confidential environment in nursing homes such as activity rooms and meeting rooms.

### Analytical approach

The data was interpreted by thematic analysis,^37,38^ with a focus on how different scripts have been incorporated into practice by assistant nurses, and why they are incorporated in the observed ways. The authors combined field notes and the interview transcripts in NVivo 12 software. To ensure the validity of the codes, member checking was used as the authors contacted with the participants to verify the interpretations of the data.^39^

To understand the incorporation of different scripts in practice, the authors first categorized the raw data into four files based on the four scripts (Figure 1). In each file, the authors then read and re-read the raw data to identify assistant nurses’ practices in relation to specific scripts. Six themes emerged (i.e. ‘receiving alarms from residents’, ‘checking received alarms via alarm phones’, ‘responding to alarms via alarm phones’, ‘checking specific residents’ situations in person’, ‘documenting all finished alarms’ and ‘documenting some finished alarms’ in Table 2).

**Table 2.** Assistant nurses’ approaches to incorporating specific technology scripts and related perceptions.

Inscribed scripts	Assistant nurses’ practices	Assistant nurses’ perceptions of incorporating the script in such a specific way	
	Themes	Sub-category	Extracted texts segments
Script 1. Alarms getting activated	Receiving alarms from residents	Reduced workload with checking residents’ situations	- No need to keep eyes on residents all the time - No need to do regular check
		Enhanced efficiency in resident information delivery	- Saved times in updating residents’ situations - Saved times in receiving residents’ demands
Script 2. Notifying designated contacts	Checking received alarms via alarm phones	Reduced workload with approaching residents	- No need to walk to residents’ rooms
		Real-time monitoring of working environments	- Can monitor more than one resident - Can monitor colleagues’ situations
		Enhanced residents’ autonomy and privacy	- Decreased frequency to disturb residents - Enhanced self-management among residents
		Disruptions in the workflows	- Have to do a series of actions to check alarms - Strict requirements for checking alarms within the 30s
		Challenges of managing all residents’ requests	- Difficult to take care of all residents - Distrust between residents and assistant nurses because of missed alarms
		Uncertainty about the ‘full picture’ of residents	- Difficult to notice residents who do not use the system
Script 3. Enabling remote communication	Responding to alarms via alarm phones	Enhanced efficiency in resident-professional communications	- Quick connections with residents - Quick recognition of residents’ needs - Quick feedbacks to residents’ needs
		Better residents’ experience in communications	- Enhanced feelings of respect and being heard among residents - Enhanced feelings of safety among residents
	Checking specific residents’ situations in person	Quiet environments for other residents	- Less noisy environments - Lower possibilities of disturbing other residents
Script 4. Tagging finished alarms	Documenting all finished alarms	A shared understanding because of synchronized alarm status	- All colleagues know which residents had received care
	Documenting some finished alarms	Reduced workload with finishing care cases	- Have to do a series of actions to tag alarms

To understand why assistant nurses conducted specific practices identified above, under each theme, the authors viewed the textual data line by line and extracted text segments regarding what assistant nurses were concerned, how they perceive the benefits or disadvantages of using the system, and how they believed their actions contribute to care provision. These extracted text segments were summarized into sub-categories according to their core meanings. To ascertain whether an extracted text segment was appropriately assigned, the authors compare text segments to segments that had been previously assigned the same sub-categories to see whether they reflected the same meaning. Through this process, the subcategories that different text segments belong to evolved. As a result, 23 extracted texts segments and thirteen sub-categories were identified. Text segments and sub-categories were reviewed by all authors to ensure consistency in the coding approach. Lastly, these text segments and subcategories were related to the identified six themes to understand how assistant nurses relate their actions, technology scripts to care provision in practice (Table 2).

### Ethical considerations

The study did not collect any personal or sensitive information and thus an ethical permission from the Ethical Review Board was not needed.^40^ Before each interview and observation, all assistant nurses were provided with an information sheet and were asked for verbal or written informed consent. The collected data were coded to ensure the involved assistant nurses’ privacy. Quotations and field notes were anonymized and reported in a way ensuring the anonymity of the involved assistant nurses.

## Results: Technology-mediated care practices in nursing homes

Table 2 outlines assistant nurses’ practices of incorporating specific technology scripts in practice and related perceptions. Briefly, the technology-mediated care practices in our study included receiving alarms from residents, checking received alarms via phones, responding to alarms via phones, checking specific residents’ situations in person, documenting all finished alarms and documenting some finished alarms.

### Receiving alarms from residents

The care practices of receiving alarms from residents were related to the script of generating and delivering alarm signals. All assistant nurses felt supported when talking about alarms delivered by the system. This script in practice contributed specifically to practical care as it reduced workload with checking residents’ situations and enhanced the efficiency in resident information delivery.

#### Reduced workload with checking residents’ situations

Assistant nurses gave examples of how they received resident information with and without the system.

AN05: It's good that you don't have to keep your eyes on them (the residents) all the time. And I know they will send alarms immediately when they need something such as a cup of coffee … Otherwise I need to do regular checks.

As described in the above quote, in the absence of the social alarm system, the assistant nurse needs to check residents’ situations regularly. The social alarm system allows her to stay passive and wait for residents’ alarms, as it can generate and deliver alarm signals from residents to assistant nurses automatically. The automatic delivery of alarm signals suggests that this script in practice decreases assistant nurses’ workloads in terms of checking residents’ situations, and thereby contributes to practical care.

#### Enhanced efficiency in resident information delivery

In terms of the care provision, there seemed to be a pervasive notion that the system increased the efficiency of information delivery between assistant nurses and residents, as this observation note revealed:

Assistant nurses need to check residents’ situations every fifteen minutes when the system is under maintenance … At 14:23, AN3 found one resident sitting on the floor. She did a regular check at 14:15 and everything was fine at that moment … ‘We could have found him earlier if he would have been able to send alarms [AN03]’

The assistant nurse made a point to highlight that the delivery of resident information without the social alarm system was not as efficient as that with the social alarm system. She believed the resident sitting on the floor could have been found earlier with the support of the social alarm system. From this perspective, the script of generating and delivering alarm signals in practice contributed to time savings for situation update and resident information delivery, especially during emergencies. Hence, the incorporation of this script in care practices is strongly related to the concept of practical care.

### Checking received alarms via alarm phones

The care practices of checking received alarms were related to the script of notifying designated contacts. All assistant nurses fully followed the script to incorporate it into their daily practices. Although the majority of assistant nurses acknowledged that checking received alarms allowed them to approach residents and recognize their needs quickly and broadly, many felt constrained because of disruptions in workflow, challenges of managing all residents’ requests and difficulties in knowing the ‘full picture’ of residents. The practices here were associated with the concepts of practical care, moral care and relational care.

#### Reduced workload with approaching residents

There was a consensus that the practice of checking received alarms benefited the reduced workload with approaching residents. For example, one assistant nurse explained how he got support from the aspect.

AN04: … They (residents) have needs, they ask (by sending alarms) … (without the system), I had to open their doors in the morning to see if they were awake and needed help.

For this assistant nurse, there is a reduction in time for meeting residents by checking received alarms. In this view, using the system helped assistant nurses to save time to approach residents. This is thus related to the concept of practical care.

#### Real-time monitoring of working environments

This same assistant nurse also mentioned that using the system allowed assistant nurses to monitor more than one resident's situation at a time and to know their colleagues’ status.

AN04: With the system, we can monitor more than one resident and know if our colleagues have checked the alarm, but before we could only check one resident at a time, and had no idea about our colleagues’ status

In this case, checking received alarms via alarm phones became a means of ensuring an equal level of care for residents and an approach to synchronizing colleagues’ status. Assistant nurses were thus empowered to understand real-time situations in their working environment more broadly. Hence, the script in practice contributed to moral care.

#### Enhanced residents’ autonomy and privacy

The two quotes above also showed the benefits of these practices to relational care. As residents would send alarms when they had needs, they have more privacy as they were less likely to be disturbed by assistant nurses during other times. In addition, residents were empowered to manage themselves and decide when to ask for help. Consequently, the practices enhanced residents’ privacy and autonomy, and thereby supported relational care.

#### Disruptions in the workflows

All assistant nurses were observed to perform similarly in response to notifications from the system. They had to either notice received alarms within 30 s, or act in a fixed way to check missed alarms. These fixed settings (i.e. limited time and required actions) left no space for assistant nurses to decide how to incorporate the script. In other words, assistant nurses had to follow the script completely in order to incorporate it into daily practices.

Many assistant nurses emphasized the disruptions in workflow caused by the required actions.

AN06: As you can see, we receive tonnes of alarms … it is challenging to deal with all the requests … It happens sometimes that they (residents) send me alarms and I am doing something much important than their requests … it is OK if we missed alarms, but we need to know what happened sooner or later … Many of us prefer to check them as soon as they come in …

According to interviews, although assistant nurses were not held accountable for not noticing alarms within 30 s, they preferred to check alarms ‘as soon as they come in’. However, an alarm could occur no matter what assistant nurses were doing and where they were. Hence, the practice of checking alarms could interrupt their ongoing work and disrupt the workflow. This was consistent with our observations, as many assistant nurses stopped their ongoing tasks to check received alarms. In this way, the script hindered care workflow, and thus challenged the provision of practical care.

#### Challenges of managing all residents’ requests

The above example also showed it was challenging for assistant nurses to manage all residents’ requests. The system enabled residents to ask for help without spatial and temporal restrictions while assistant nurses may be ‘doing something much important’. In our observations, some residents complained about the time for waiting for responses, and showed that they did not believe assistant nurses were that busy to ignore their alarms. In this regard, the script in practice inhibited relational care because of the increased difficulties in managing residents’ requests and the emerged distrust between residents and assistant nurses.

#### Uncertainty about the ‘full picture’ of residents

In addition to the disruptions in workflow and challenges of managing all residents’ requests, a few assistant nurses expressed concern about knowing ‘the full picture’ of residents. Given the fact that residents who sent alarms received more attention, this group of assistant nurses worried that ‘shy’ residents may not be cared for adequately.

AN05: … some residents are out of the system. Like [PT04], one morning I noticed that she didn't get up as usual. It turned out that she was having toileting difficulties but was too shy to ask for help. For one hour, she sat there. She is out of the system … we should not rely on the received alarms to care for people like her. We may lose the full picture of residents.

The above case communicated a tension the assistant nurses felt between the residents’ actual situations and the situation conveyed via social alarm system information. For residents who were not used to asking for help through the social alarm system, their needs were ‘out of the system’. Hence, these residents would receive less attention if assistant nurses provided care based on the received alarms. Relying on notifications from the system could thus enhance care accessibility for residents who are actively engaged in using the system, whereas care accessibility could be inhibited for those residents who are less active. The social alarm system accordingly has the potential to widen inequities in care access, an aspect which belongs to moral care.

### Responding to alarms via alarm phones

The care practices of responding to alarms via alarm phones were related to the script of enabling remote communication. Totally following the script in practice, assistant nurses believe that their practices benefit the efficiency in resident-professional communications and residents’ experiences. Practical care and relational care were greatly considered in related cases.

#### Enhanced efficiency in resident-professional communications

As one assistant nurse mentioned, with a simple click, she was able to talk with residents remotely.

AN06 stopped cooking and checked the received alarm. She then clicked the ‘respond’ button and talked to [PT 07]: ‘Sure, I will do it now’. She prepared a cup of coffee and sent it to [PT07]’s room … ‘Who would refuse it (responding to alarms)’, AN06 laughed, ‘It helps to know what they (residents) want in detail quickly … They send you alarms, they expect your reply promptly … calling back through the system is convenient, and makes them feel that they are being heard’.

In this case, the assistant nurse referenced ‘responding to alarms via phones’ as convenient in terms of connecting to specific residents, understanding detailed resident needs, and giving quick feedbacks. Talking to the resident via the social alarm system allowed her to break the spatial boundaries with the resident, and to enhance the efficiency in communicating with residents. The time saved here was related to the concept of practical care.

#### Better residents’ experience in communications

The case above also pointed out the benefits of responding to alarms in calming down residents. The assistant nurse highlighted that the remote communication enabled prompt replies to residents, which eased their minds. The emphasis on better residents’ feelings suggested that care practices here contributed to relational care.

### Checking specific residents’ situations in person

The care practices of checking specific residents’ situations in person were also related to the script of enabling remote communication. During our observations, assistant nurses sometimes unfollowed the script in practice as they thought responding to alarms via phones may lead to noisy environments that disturbing other residents. Moral care was considered under this context.

#### Quiet environments for other residents

Rather than responding to received alarms via alarm phones, the same assistant nurses in the example above sometimes went to the residents’ rooms directly after checking the received alarms. In this way, the script of enabling remote communication was abandoned. This kind of behaviour was also observed among the other assistant nurses, especially in the afternoon. The same assistant nurse gave her explanations during the interview:

AN06: Some residents are used to taking a nap after lunch. Talking on the phone may disturb those residents who are sleeping.

The assistant nurse changed the approach of incorporating the script due to her concerns about the other residents. For her, the remote communication might disturb other residents who needed a quiet environment. In addition to the resident who sent an alarm, she also cared about the other residents in the nursing home. The provision of moral care was greatly considered under such a context.

### Documenting all finished alarms

The care practices of documenting all finished alarms were related to the script of tagging finished alarms. Assistant nurses totally follow the script in practice, which they perceive as beneficial to a shared understanding of the working environments. Here, practical care and moral care are supported.

#### A shared understanding because of synchronized alarm status

Take, for example, the following assistant nurse, she totally followed the script of tagging finished alarms in her practice:

AN10 finished her tasks for [PT14]. She unlocked the screen, tapped the alarm sent by [PT14], and tapped the ‘finished’ button. She showed me the screen: ‘It is important to do this. The status of alarms is synchronized in all alarm phones, so my colleagues can see the update too. The list is clear now. With one glance, all of us can know which alarms have been finished or are unfinished’.

She explained that following the script, in this case, would contribute to a clear alarm list, which helped her and her colleagues to recognize unfinished care tasks quickly. The time used for information sharing among colleagues was saved. Besides, all assistant nurses have a deeper understanding of what is happening in the nursing homes. Therefore, the practices of documenting all finished alarms supported practical care and moral care.

### Documenting some finished alarms

Besides totally following the script, assistant nurses were observed to selectively document finished alarms. Contradictory perspectives were observed, as some assistant nurses believed it did not worth the time and energy to document all alarms. Despite the different behaviours in dealing with the finished alarms, all assistant nurses considered the concepts of practical care and moral care.

#### Reduced workload with finishing care cases

While the assistant nurse above felt empowered by the practices of documenting all finished alarms, some assistant nurses held a different idea. They believed that there was no need to document every finished alarm. Though acknowledging the importance of a clear alarm list, they selectively documented finished alarms.

AN12 finished [PT22]’s case but did not document the corresponding alarm. She then went to [PT23]’s room directly. She tagged [PT23]’s alarm as ‘finished’ later on … ‘When I was caring for [PT22], my colleagues were with me. So they knew the case had been handled. I know we need it (alarm list) to be easy to understand. But it does not worth spending time and energy documenting this one’.

The assistant nurse above framed documenting the resident's alarm as a worthless endeavour and refused to document the alarm from [PT22] as other colleagues had known related situations. Her comments suggested that assistant nurses tended to abandon the script when its expected impact (e.g. colleagues knew which resident had received care) had been achieved. Despite the different approaches to incorporating the script of tagging finished alarms, the assistant nurse mentioned the time and energy in providing care. Specifically, practical care was constrained as the practices of documenting all finished alarms were viewed as time-consuming and energy-consuming.

## Discussion: Re-thinking the technology scripts in care practices

Drawing on the concept of technology scripts, the study specified assistant nurses’ related technology-mediated care practices, including receiving alarms from residents, checking received alarms via alarm phones, responding to alarms via alarm phones, checking specific residents’ situations in person, documenting all finished alarms and documenting some finished alarms. Within each practice, specific technology scripts have complex relationships with different care concepts. In line with current studies, the findings indicate that using technology in practice is a non-linear process and is something more than simply placing the devices in targeted places.^11,41–43^ This article further adds to this knowledge by elucidating how different instances and concepts introduced by the system are incorporated into care practices that were overflowing with different care concepts (Table 3).

**Table 3.** Technology scripts in assistant nurses’ practices and their relations with different care concepts.

Inscribed scripts	Assistant nurses’ practices and perceptions	Effects on care practices
Script 1. Alarms getting activated	Following the script as it - reduced workload with checking residents’ situations - enhanced efficiency in resident information delivery	Support - practical care
Script 2. Notifying designated contacts	Following the script as it - reduced workload with approaching residents - benefited the real-time monitoring of working environments - enhanced residents’ autonomy and privacy But it also bought issues about - disruptions in the workflows - challenges of managing all residents’ requests - uncertainty about the ‘full picture’ of residents	Support - practical care - moral care - relational care Inhibit - practical care - moral care - relational care
Script 3. Enabling remote communication	Following the script as it - enhanced efficiency in resident-professional communications - enabled better residents’ experience in communications Unfollowing the script for - quiet environments for other residents	Support - practical care - relational care Inhibit - moral care
Script 4. Tagging finished alarms	Following the script as it - enabled a shared understanding because of synchronized alarm status better Unfollowing the script as it - reduced workload with finishing care cases	Support - practical care - moral care Inhibit - practical care

### Different relationships between the technology scripts and care concepts in practice

Our results highlighted the different relationships between the technology scripts and care concepts. When incorporated into care practices, some scripts may enhance care outcomes in relation to specific care concepts, some may enhance and constrain the ones in relation to the same care concepts at the same time, and some may enhance care outcomes in relation to certain care concepts while inhibiting the ones in relation to other care concepts. For instance, the script of notifying designated contacts is associated with the concept of practical care, relational care and moral care (Table 3). While it can enhance care outcomes from the three dimensions of care by increasing the efficiency and range of recognizing residents’ needs and colleagues’ status, the script in practice would also threaten care outcomes about the three care concepts because of potential disruptions in workflow, emerging challenges of dealing with all residents’ requests and risks of ignoring ‘the full picture’ of residents. The script of enabling remote communication in practice is an example to show how the same script may enhance care outcomes in relation to specific care concepts while threatening the ones in relation to other care concepts. Although the practices of checking received alarms via phones support practical care and relational care because of the benefits of enhanced communication efficiency and better residents’ experience, they inhibit moral care as responding to alarms via phones may disturb those who need a quiet environment.

The different relationships presented above illustrate how even a simple technology can bring both possibilities and challenges when being integrated into care practices, and how its inscribed scripts may support or inhibit care provision from different dimensions. The findings add to the current discussion that technology integration should be viewed critically, with a focus on both potential benefits and risks.

### Assistant nurses’ divergent actions of (un)following specific scripts

As described, technology scripts that are perceived as beneficial might, in fact, threaten care provision in certain dimensions. According to our observations, assistant nurses may follow specific scripts in certain cases. However, they may also unfollow the same scripts under different contexts. The cases of responding to alarms via phones are quite revealing in this aspect. The related script, enabling remote voice communication, expects that the remote communication starts after assistant nurses receive residents’ alarms. However, in our cases, the same assistant nurse may decide to respond to alarms via the system or have face-to-face communication with the residents in different contexts. Besides, despite the same contexts, different assistant nurses may have different approaches to incorporating the same script in practice. For example, when incorporating the script of tagging finished alarms, some assistant nurses document all finished alarms whereas some only document some finished alarms.

Our findings reveal how unpredictable is the incorporation of technology scripts in practice. It is impossible to completely predict how the actors involved incorporate specific technology scripts in care practices, and what are the impacts that entails. This is because of the complexity of different contexts and the diverse approaches that are used by actors involved.

### Assistant nurses’ situational and personal interpretations of scripts in practice

Having recognized the complexity of technology incorporation in care practices, the question of how assistant nurses deal with the technology scripts in practice arises. The findings reveal that assistant nurses decide their approaches to incorporating specific scripts in practice based on their situational and personal interpretations.

Specifically, in our cases, assistant nurses defined the technology scripts as they allocated different care concepts to the scripts based on the scripts’ expected requirements and outcomes. This is in line with recent studies arguing that technologies are not value-neutral.^44^ For example, the script of tagging finished alarms expects assistant nurses to conduct particular actions to document finished alarms (e.g. unlock the phone screens, click particular buttons etc.). By doing so, the system is able to present a clear alarm list, and thus to ensure real-time updated environmental information (e.g. colleagues’ status, finished care cases etc.) among all assistant nurses. However, some assistant nurses complained about the actions required for documenting alarms, referencing these as ‘not worth spending time and energy’ in certain cases. On the other hand, some assistant nurses acknowledged the benefits of the created alarm list as it allowed all of them to update real-time environmental information. The script of tagging finished alarms here appears to enhance the moral care due to the contributions to real-time information updates, whereas it constrains the practical care because of the required ‘worthless’ time and energy. That is, assistant nurses, by expressing personal thoughts on the script's expected requirements and outcomes, attribute particular care concepts to the considered technology scripts.

In parallel with defining the technology scripts, assistant nurses considered the quality of care by evaluating the situations at hand, and prioritizing different care concepts based on these. When the scripts’ care concepts were consistent with the high-priority care concepts, the scripts were more likely to function as expected. Otherwise, assistant nurses may either abandon the scripts or adjust their ways of incorporating different scripts into practice in order to prioritize the high-priority care concepts. The script of signal generation and delivery, for example, aims to enhance the efficiency of resident information delivery, and thus supports both practical care and relational care. One case observed in the study was that one resident was found sitting on the floor for a while, without a channel to ask for help. The interviewed assistant nurse believed that she ‘could have found him earlier if would have been able to send alarms’. In this situation, the assistant nurse prioritized the concept of practical care, as she highlighted the importance of saving time in receiving the resident's requests. Given that the high-priority care concept was consistent with the script's care concepts, in this case, the script of generating and delivering alarm signals was thus appreciated and followed.

Taking a closer look at assistant nurses’ interpretations, rather than adhering to the procedures or rules of technology use or certain care concepts, assistant nurses tend to identify the right thing to do in specific situations. In this view, what matters to them is not whether the system is used in a predetermined way, or whether using the technology has achieved its expected impacts, but rather how care should be provided in specific situations where technology, individual actions and approaches, and care concepts are mixed together. This explains why technology scripts are not incorporated as expected in our study. Current studies indicate that the unexpected use of technology is impressive because it shows users’ vitality and imagination, but that it is also thought to be useless or unsafe because it can prevent well-designed systems from complying with standards of safety and quality.^2,4,11,45^ Our study considered this question from a perspective of care provision, and answered this question by saying that the unexpected use of technology may symbolize a strategy of caring for residents in a specific situation, rather than ignorance or negligence to adhere to standards of safety and quality. In this regard, we believe that understanding the care practices in specific situations is a good starting point from which to provide recommendations that are more sensitive to the complexities of technology-mediated care work.

## Limitations

This study had some limitations. First, given our small number of participants, there may be other situations that were not observed. Additionally, the diversity of participants should be considered as it may have affected the findings. As described, because the collected data focused entirely on assistant nurses’ experiences and narratives, our results and discussions are limited to the care work from the assistant nurses’ perspectives. We understand the importance of a diverse participant group. Future research should thereby include other stakeholders, such as managers and residents, in order to understand the full map of care work in nursing homes.

## Conclusions

This study reveals the complexity of incorporating technology scripts into care practices overflowing with different care concepts. The results illustrate the characteristics of technology scripts in care practices, how technology scripts become (or do not become) part of assistant nurses’ care practices, and how these scripts are interpreted in relation to the quality of care. This study makes a two-fold contribution. First, it extends the theme on the script concept, as the current literature mainly focuses on private settings (e.g. older adults’ homes) rather than on institutional settings (e.g. nursing homes), and pays little attention to how the script concept in these settings relates to different care concepts in practice. Second, it elaborates on how technology-mediated care practices are being delivered as practical accomplishments for care provision. This could be of use to implementers, technology designers, and researchers to help understand the complex reality of technology in use in care practice.
